# Genomic Assemblies of Members of *Burkholderia* and Related Genera as a Resource for Natural Product Discovery

**DOI:** 10.1128/MRA.00485-20

**Published:** 2020-10-15

**Authors:** Alex J. Mullins, Cerith Jones, Matthew J. Bull, Gordon Webster, Julian Parkhill, Thomas R. Connor, James A. H. Murray, Gregory L. Challis, Eshwar Mahenthiralingam

**Affiliations:** a Microbiomes, Microbes, and Informatics Group, School of Biosciences, Cardiff University, Cardiff, United Kingdom; b Wellcome Sanger Institute, Cambridge, United Kingdom; c Molecular Biosciences Division, School of Biosciences, Cardiff University, Cardiff, United Kingdom; d Department of Chemistry, University of Warwick, Coventry, United Kingdom; e Warwick Integrative Synthetic Biology Centre, University of Warwick, Coventry, United Kingdom; f Department of Biochemistry and Molecular Biology, Monash University, Clayton, Victoria, Australia; University of Maryland School of Medicine

## Abstract

The genomes of 450 members of *Burkholderiaceae*, isolated from clinical and environmental sources, were sequenced and assembled as a resource for genome mining. Genomic analysis of the collection has enabled the identification of multiple metabolites and their biosynthetic gene clusters, including the antibiotics gladiolin, icosalide A, enacyloxin, and cepacin A.

## ANNOUNCEMENT

*Burkholderia* is a diverse genus of bacteria and a rich source of antimicrobial compounds (1). Modern natural product discovery is often guided by genome mining (2) and increasingly relies on large genome collections. Therefore, we have sequenced, assembled, and deposited into public databases a substantial collection of genomes of members of *Burkholderia* and related genera to promote natural product discovery.

Genomic DNA was extracted, using the automated Maxwell 16 instrument with the tissue extraction kit (Promega), from *Burkholderia* strains and strains of related genera that had been grown in tryptic soy broth overnight at 30°C or 37°C. HiSeq 2000 and HiSeq X Ten instruments, with the associated Illumina HiSeq kits for library preparation, generated 125-bp and 150-bp paired-end reads, respectively. Reads were trimmed using Trim Galore v0.4.2 (3), their quality was assessed using FastQC v0.10.1 (4), and overlapping reads were merged using FLASH v1.2.11 (5). The resulting reads were assembled into contigs using the assembler SPAdes v3.9.1 (6). Pilon v1.2125 (7) identified and corrected misassembled contigs, and contigs representing contaminating DNA were identified with Kraken v0.10.5-beta (8) using the MiniKraken database. In addition, four *Burkholderia* strains were sequenced with a Pacific Bioscience RS II instrument using genomic DNA prepared with the SMRTbell template preparation kit v2.0. The sequencing was performed in two runs with P4-C2 and P5-C3 chemistries, and the genomes were assembled with SMRT Analysis v2.3.0 as described previously (9). A quality assessment of the final genome assemblies was performed with QUAST v4.4 (10) (Table 1). Genomic identification was performed by average nucleotide identity (ANI) analyses with fastANI v1.2 (11) and pyani v0.2.9 (ANI using MUMmer [ANIm] to align input sequences) (12), using a ≥95% species delineation threshold (11). The NCBI Prokaryotic Genome Annotation Pipeline (PGAP) v4.12 was used for genome annotation (13). Bioinformatics were supported by the Cloud Infrastructure for Microbial Bioinformatics (CLIMB) (14). Default software parameters were used except where otherwise noted.

**TABLE 1:** Strain information and genome assembly statistics for the *Burkholderiaceae* genome collection

Species	Strain	Isolation source	Geographic location	Read BioSample accession no.	Assembly BioSample accession no.	Assembly GenBank accession no.	Coverage (×) (technology)	Genome size (Mbp)	GC content (%)	*N*_50_ (bp)
*Burkholderia ambifaria*	BCC0118	Clinical, sputum	USA	ERS784989	ERS4267623	GCA_902829755	81 (Illumina)	7.50	66.82	273,879
*Burkholderia ambifaria*	BCC0191	Environmental, soil	USA	ERS784799	ERS4267624	GCA_902829465	82 (Illumina)	7.58	66.56	302,202
*Burkholderia ambifaria*	BCC0192	Environmental	USA	ERS785047	ERS4267625	GCA_902829785	73 (Illumina)	7.40	66.73	277,504
*Burkholderia ambifaria*	BCC0197	Environmental, *Sesbania exaltata* leaves		ERS785076	ERS4267626	GCA_902829415	71 (Illumina)	7.38	66.67	386,867
*Burkholderia ambifaria*	BCC0250	Clinical, sputum	Australia	ERS784819	ERS4267627	GCA_902829765	86 (Illumina)	7.36	66.84	396,147
*Burkholderia ambifaria*	BCC0267	Clinical, cystic fibrosis patient	Australia	ERS784835	ERS4267628	GCA_902829645	80 (Illumina)	7.36	66.76	784,052
*Burkholderia ambifaria*	BCC0284	Environmental, corn	USA	ERS1328916	ERS4267629	GCA_902829725	59 (Illumina)	7.47	66.68	423,851
*Burkholderia ambifaria*	BCC0316	Environmental, soil	USA	ERS784850	ERS4267630	GCA_902829685	83 (Illumina)	7.64	66.54	292,321
*Burkholderia ambifaria*	BCC0338	Environmental, corn roots	USA	ERS1371637	ERS4267631	GCA_902829825	90 (Illumina)	7.45	66.70	514,723
*Burkholderia ambifaria*	BCC0399	Environmental	USA	ERS785037	ERS4267632	GCA_902829775	77 (Illumina)	7.40	66.76	407,605
*Burkholderia ambifaria*	BCC0410	Environmental, maize	Italy	ERS784866	ERS4267633	GCA_902829705	93 (Illumina)	7.38	66.78	849,830
*Burkholderia ambifaria*	BCC0423	Environmental, maize	Italy	ERS1336067	ERS4267634	GCA_902829805	73 (Illumina)	7.47	66.76	382,759
*Burkholderia ambifaria*	BCC0477	Clinical, cystic fibrosis patient	USA	ERS784882	ERS4267635	GCA_902829695	99 (Illumina)	7.81	66.47	255,598
*Burkholderia ambifaria*	BCC0478	Clinical, cystic fibrosis patient	USA	ERS784897	ERS4267636	GCA_902829665	109 (Illumina)	7.24	66.82	601,161
*Burkholderia ambifaria*	BCC0480	Environmental, soil	USA	ERS784913	ERS4267637	GCA_902829735	113 (Illumina)	7.84	66.57	570,779
*Burkholderia ambifaria*	BCC1041	Environmental	Italy	ERS784930	ERS4267638	GCA_902829815	104 (Illumina)	7.51	66.70	469,567
*Burkholderia ambifaria*	BCC1048	Environmental	Italy	ERS784886	ERS4267639	GCA_902829675	70 (Illumina)	7.51	66.70	469,249
*Burkholderia ambifaria*	BCC1052	Environmental	Italy	ERS1371632	ERS4267640	GCA_902829435	72 (Illumina)	7.33	66.81	397,259
*Burkholderia ambifaria*	BCC1062	Environmental	Italy	ERS1328829	ERS4267641	GCA_902829655	65 (Illumina)	7.45	66.69	382,455
*Burkholderia ambifaria*	BCC1065	Environmental	Italy	ERS784959	ERS4267642	GCA_902829835	77 (Illumina)	7.31	66.79	605,534
*Burkholderia ambifaria*	BCC1066	Environmental	Italy	ERS784800	ERS4267643	GCA_902829905	87 (Illumina)	7.32	66.78	381,533
*Burkholderia ambifaria*	BCC1072	Environmental	Italy	ERS1328913	ERS4267644	GCA_902830005	66 (Illumina)	7.44	66.75	397,405
*Burkholderia ambifaria*	BCC1080	Environmental	Italy	ERS1371635	ERS4267645	GCA_902829995	84 (Illumina)	7.29	66.76	383,339
*Burkholderia ambifaria*	BCC1083	Environmental	Italy	ERS1328835	ERS4267646	GCA_902829965	63 (Illumina)	7.30	66.76	373,328
*Burkholderia ambifaria*	BCC1086	Environmental	Italy	ERS1328827	ERS4267647	GCA_902829915	70 (Illumina)	7.58	66.72	395,516
*Burkholderia ambifaria*	BCC1088	Environmental	Italy	ERS1371633	ERS4267648	GCA_902829875	87 (Illumina)	7.48	66.70	397,405
*Burkholderia ambifaria*	BCC1090	Environmental	Italy	ERS1328833	ERS4267649	GCA_902829975	68 (Illumina)	7.33	66.76	340,920
*Burkholderia ambifaria*	BCC1092	Environmental	Italy	ERS784808	ERS4267650	GCA_902829935	77 (Illumina)	7.38	66.78	849,686
*Burkholderia ambifaria*	BCC1093	Environmental	Italy	ERS784821	ERS4267651	GCA_902829945	81 (Illumina)	7.40	66.76	427,669
*Burkholderia ambifaria*	BCC1095	Environmental	Italy	ERS784837	ERS4267652	GCA_902829885	84 (Illumina)	7.40	66.76	407,577
*Burkholderia ambifaria*	BCC1098	Environmental	Italy	ERS784852	ERS4267653	GCA_902829895	83 (Illumina)	7.38	66.78	849,506
*Burkholderia ambifaria*	BCC1100	Environmental	Italy	ERS784868	ERS4267654	GCA_902830015	80 (Illumina)	7.40	66.76	407,581
*Burkholderia ambifaria*	BCC1103	Environmental	Italy	ERS784884	ERS4267655	GCA_902829985	86 (Illumina)	7.40	66.76	427,387
*Burkholderia ambifaria*	BCC1105	Environmental	Italy	ERS1371636	ERS4267656	GCA_902829845	97 (Illumina)	6.30	66.77	1,592,784
*Burkholderia ambifaria*	BCC1107	Environmental	Italy	ERS784899	ERS4267657	GCA_902829855	88 (Illumina)	7.40	66.76	374,059
*Burkholderia ambifaria*	BCC1212	Environmental, rhizosphere	USA	ERS1371638	ERS4267658	GCA_902829955	82 (Illumina)	7.61	66.44	358,611
*Burkholderia ambifaria*	BCC1213	Environmental	USA	ERS1328957	ERS4267659	GCA_902829925	65 (Illumina)	7.47	66.70	425,516
*Burkholderia ambifaria*	BCC1214	Environmental	USA	ERS784915	ERS4267660	GCA_902829595	102 (Illumina)	7.61	66.40	270,842
*Burkholderia ambifaria*	BCC1216	Environmental	USA	ERS1328918	ERS4267661	GCA_902829865	67 (Illumina)	7.46	66.69	234,327
*Burkholderia ambifaria*	BCC1218	Environmental	USA	ERS784932	ERS4267662	GCA_902830025	88 (Illumina)	7.46	66.67	348,377
*Burkholderia ambifaria*	BCC1220	Environmental	USA	ERS1328837	ERS4267663	GCA_902830035	65 (Illumina)	7.64	66.39	393,412
*Burkholderia ambifaria*	BCC1223	Environmental	USA	ERS784947	ERS4267664	GCA_902830105	104 (Illumina)	7.61	66.40	267,383
*Burkholderia ambifaria*	BCC1224	Environmental, maize	USA	ERS1328836	ERS4267665	GCA_902829395	51 (Illumina)	8.46	63.24	317,020
*Burkholderia ambifaria*	BCC1228	Environmental, maize	USA	ERS1328832	ERS4267666	GCA_902830065	62 (Illumina)	7.38	66.73	558,864
*Burkholderia ambifaria*	BCC1229	Environmental, maize	USA	ERS1371639	ERS4267667	GCA_902830045	65 (Illumina)	7.51	66.70	298,531
*Burkholderia ambifaria*	BCC1233	Environmental, maize	USA	ERS1328917	ERS4267668	GCA_902830075	57 (Illumina)	7.96	66.24	390,415
*Burkholderia ambifaria*	BCC1236	Environmental, maize	USA	ERS1328839	ERS4267669	GCA_902830095	64 (Illumina)	7.63	66.60	358,767
*Burkholderia ambifaria*	BCC1237	Environmental, maize	USA	ERS1371640	ERS4267670	GCA_902830055	66 (Illumina)	7.34	66.84	420,767
*Burkholderia ambifaria*	BCC1240	Environmental, maize	USA	ERS1328914	ERS4267671	GCA_902830085	57 (Illumina)	7.40	66.77	503,900
*Burkholderia ambifaria*	BCC1241	Environmental, maize	USA	ERS784961	ERS4267672	GCA_902830175	78 (Illumina)	7.68	66.49	288,780
*Burkholderia ambifaria*	BCC1246	Environmental, maize	USA	ERS1328834	ERS4267673	GCA_902830205	69 (Illumina)	7.48	66.64	450,462
*Burkholderia ambifaria*	BCC1248	Environmental, maize	USA	ERS784801	ERS4267674	GCA_902830135	87 (Illumina)	8.03	66.48	241,997
*Burkholderia ambifaria*	BCC1249	Environmental, maize	USA	ERS1371634	ERS4267675	GCA_902830255	97 (Illumina)	7.58	66.75	447,443
*Burkholderia ambifaria*	BCC1252	Environmental, maize	USA	ERS784809	ERS4267676	GCA_902830125	83 (Illumina)	7.42	66.71	254,705
*Burkholderia ambifaria*	BCC1256	Environmental, maize	USA	ERS784823	ERS4267677	GCA_902830165	86 (Illumina)	7.54	66.60	281,063
*Burkholderia ambifaria*	BCC1258	Environmental, maize	USA	ERS1371641	ERS4267678	GCA_902829475	81 (Illumina)	7.60	66.61	272,850
*Burkholderia ambifaria*	BCC1259	Environmental, maize	USA	ERS784838	ERS4267679	GCA_902830225	78 (Illumina)	7.63	66.39	431,727
*Burkholderia ambifaria*	BCC1265	Environmental	USA	ERS784854	ERS4267680	GCA_902830115	79 (Illumina)	7.62	66.44	335,885
*Burkholderia ambifaria*	BCC1270	Environmental, maize	USA	ERS784870	ERS4267681	GCA_902830155	73 (Illumina)	7.60	66.61	315,858
*Burkholderia multivorans*	BCC1599	Environmental		ERS1371642	ERS4267682	GCA_902833025	87 (Illumina)	6.74	67.15	248,547
*Burkholderia* sp.	BCC1630	Environmental	Malaysia	ERS1328847	ERS4267683	GCA_902833165	75 (Illumina)	7.62	66.03	201,973
*Burkholderia* sp.	BCC1638	Environmental	Malaysia	ERS1328851	ERS4267684	GCA_902833195	89 (Illumina)	6.96	66.61	206,632
*Burkholderia anthina*	BCC0036	Clinical, cystic fibrosis patient	UK	ERS785067	ERS4267685	GCA_902830145	69 (Illumina)	7.72	66.49	216,836
*Burkholderia anthina*	BCC0403	Environmental, river	USA	ERS784934	ERS4267686	GCA_902830325	76 (Illumina)	8.15	66.41	375,294
*Burkholderia anthina*	BCC0485	Clinical, cystic fibrosis patient	USA	ERS1328919	ERS4267687	GCA_902829375	49 (Illumina)	9.30	61.27	152,611
*Burkholderia anthina*	BCC0518	Environmental, soil	UK	ERS1328922	ERS4267688	GCA_902830485	66 (Illumina)	8.28	66.46	220,529
*Burkholderia anthina*	BCC0634	Environmental, soil rhizosphere	USA	ERS1328841	ERS4267689	GCA_902830375	87 (Illumina)	7.94	65.74	168,505
*Burkholderia anthina*	BCC0635	Environmental, rhizosphere		ERS1333586	ERS4267690	GCA_902830445	89 (Illumina)	7.76	66.03	194,880
*Burkholderia anthina*	BCC0636	Environmental, *Carludovica palmata* rhizosphere	UK	ERS1328842	ERS4267691	GCA_902830345	95 (Illumina)	7.76	66.03	195,048
*Burkholderia anthina*	BCC0637	Environmental, soil		ERS1328920	ERS4267692	GCA_902830315	101 (Illumina)	7.30	66.36	205,801
*Burkholderia anthina*	BCC0638	Environmental, soil		ERS1328843	ERS4267693	GCA_902829625	102 (Illumina)	7.69	66.33	147,404
*Burkholderia anthina*	LMG 20980	Environmental, soil rhizosphere	USA	ERS1371643	ERS4267694	GCA_902830395	71 (Illumina)	7.63	66.74	336,735
*Burkholderia anthina*	BCC1349	Clinical		ERS1328844	ERS4267695	GCA_902830335	76 (Illumina)	8.61	66.32	444,914
*Burkholderia stagnalis*	BCC1350	Environmental		ERS784963	ERS4267696	GCA_902833065	83 (Illumina)	8.37	67.13	251,925
*Burkholderia anthina*	BCC1351	Clinical		ERS1328921	ERS4267697	GCA_902829335	94 (Illumina)	7.48	66.21	196,378
*Burkholderia anthina*	BCC1358	Clinical		ERS1371644	ERS4267698	GCA_902830365	68 (Illumina)	9.16	66.08	173,669
*Burkholderia anthina*	BCC1363	Environmental		ERS1336069	ERS4267699	GCA_902830415	85 (Illumina)	7.28	66.32	170,879
*Burkholderia arboris*	BCC0049	Clinical, non-cystic fibrosis patient	France	ERS785032	ERS4267700	GCA_902830425	64 (Illumina)	8.28	66.77	418,964
*Burkholderia cenocepacia*	J2315	Clinical, respiratory sample	UK	ERS784840	ERS4267701	GCA_902830575	82 (Illumina)	7.91	66.99	221,803
*Burkholderia cenocepacia*	BCC0016	Clinical, sputum	Canada	ERS784856	ERS4267702	GCA_902830355	81 (Illumina)	7.89	67.01	175,882
*Burkholderia cenocepacia*	BCC0017	Clinical, sputum	Canada	ERS785082	ERS4267703	GCA_902830435	79 (Illumina)	8.10	67.17	243,601
*Burkholderia cepacia*	BCC0018	Clinical, cystic fibrosis patient	UK	ERS785027	ERS4267704	GCA_902830685	59 (Illumina)	8.54	66.76	891,303
*Burkholderia cenocepacia*	BCC0020	Clinical, cystic fibrosis patient	Australia	ERS784871	ERS4267705	GCA_902830475	88 (Illumina)	7.30	66.88	536,526
*Burkholderia cenocepacia*	BCC0048	Clinical, non-cystic fibrosis patient	France	ERS784998	ERS4267706	GCA_902830385	64 (Illumina)	8.32	66.91	293,098
*Burkholderia* sp.	BCC0097	Clinical, respiratory sample	Canada	ERS784888	ERS4267707	GCA_902832985	65 (Illumina)	7.94	66.71	613,508
*Burkholderia cenocepacia*	BCC0107	Clinical, sputum	Canada	ERS785010	ERS4267708	GCA_902830455	68 (Illumina)	8.19	67.10	293,110
*Burkholderia cenocepacia*	BCC0127	Clinical, sputum	USA	ERS785018	ERS4267709	GCA_902830405	71 (Illumina)	7.73	66.79	420,157
*Burkholderia contaminans*	BCC0132	Clinical, sputum	Canada	ERS785057	ERS4267710	GCA_902830775	64 (Illumina)	8.46	66.61	151,177
*Burkholderia cenocepacia*	BCC0138	Clinical	Canada	ERS785048	ERS4267711	GCA_902830465	67 (Illumina)	7.99	67.24	228,664
*Burkholderia cenocepacia*	BCC0160	Clinical	USA	ERS784903	ERS4267712	GCA_902830505	81 (Illumina)	6.98	66.93	421,783
*Burkholderia cenocepacia*	BCC0165	Clinical, cystic fibrosis patient	Belgium	ERS785066	ERS4267713	GCA_902830495	73 (Illumina)	7.52	66.96	1,954,875
*Burkholderia cenocepacia*	BCC0199	Environmental	USA	ERS785064	ERS4267714	GCA_902830545	72 (Illumina)	7.97	66.66	275,726
*Burkholderia cenocepacia*	BCC0202	Environmental	USA	ERS784992	ERS4267715	GCA_902830535	84 (Illumina)	7.61	66.87	596,170
*Burkholderia cenocepacia*	BCC0313	Clinical		ERS784920	ERS4267716	GCA_902830515	77 (Illumina)	8.62	65.53	301,741
*Burkholderia contaminans*	BCC0320	Environmental, radish	Mexico	ERS784936	ERS4267717	GCA_902830835	84 (Illumina)	8.33	66.35	606,600
*Burkholderia cenocepacia*	BCC0451	Environmental, maize		ERS784950	ERS4267718	GCA_902830565	91 (Illumina)	7.52	66.92	1,207,947
*Burkholderia* sp.	BCC0506	Clinical, cystic fibrosis patient	Italy	ERS784858	ERS4267719	GCA_902833145	90 (Illumina)	7.29	67.22	506,111
*Burkholderia* sp.	BCC0801	Clinical, cystic fibrosis patient	Argentina	ERS784873	ERS4267720	GCA_902833175	88 (Illumina)	7.50	67.06	298,805
*Burkholderia cenocepacia*	BCC0999	Environmental		ERS784803	ERS4267721	GCA_902830585	96 (Illumina)	7.62	66.81	225,632
*Burkholderia cenocepacia*	BCC1028	Environmental		ERS784811	ERS4267722	GCA_902830605	91 (Illumina)	7.59	66.87	470,293
*Burkholderia* sp.	BCC1208	Environmental, lupine shoot endophyte	Australia	ERS784827	ERS4267723	GCA_902833095	91 (Illumina)	7.38	66.96	490,408
*Burkholderia cenocepacia*	BCC1266	Environmental		ERS785055	ERS4267724	GCA_902830595	65 (Illumina)	7.92	66.65	259,121
*Burkholderia cenocepacia*	BCC1322	Environmental/industrial		ERS784842	ERS4267725	GCA_902829445	82 (Illumina)	7.94	66.75	571,121
*Burkholderia cenocepacia*	BCC1556	Environmental/industrial	UK	ERS785001	ERS4267726	GCA_902830525	78 (Illumina)	7.21	67.13	1,136,705
*Burkholderia cepacia*	BCC1631	Environmental		ERS1328961	ERS4267727	GCA_902830715	71 (Illumina)	8.72	66.64	276,956
*Burkholderia cepacia*	BCC1632	Environmental		ERS1328848	ERS4267728	GCA_902830785	83 (Illumina)	8.83	66.59	273,961
*Burkholderia cepacia*	BCC1634	Environmental		ERS1328849	ERS4267729	GCA_902830765	90 (Illumina)	7.59	66.62	403,579
*Burkholderia cepacia*	BCC1636	Environmental		ERS1328850	ERS4267730	GCA_902829425	88 (Illumina)	8.48	66.70	266,835
*Burkholderia cepacia*	BCC1641	Environmental	Malaysia	ERS1328925	ERS4267731	GCA_902830805	80 (Illumina)	8.72	66.64	256,085
*Burkholderia* sp.	BCC1644	Environmental		ERS1371646	ERS4267732	GCA_902833225	71 (Illumina)	8.25	66.71	390,260
*Burkholderia cenocepacia*	BCC1718	Clinical		ERS785000	ERS4267733	GCA_902830555	70 (Illumina)	7.70	66.81	543,487
*Burkholderia cepacia*	BCC0001	Environmental, onion		ERS784996	ERS4267734	GCA_902830625	67 (Illumina)	8.51	66.65	467,216
*Burkholderia cepacia*	BCC0002	Environmental, forest soil	Trinidad and Tobago	ERS784890	ERS4267735	GCA_902830635	68 (Illumina)	8.54	66.76	891,307
*Burkholderia cepacia*	BCC0003	Clinical, cystic fibrosis patient	Australia	ERS784995	ERS4267736	GCA_902830665	68 (Illumina)	8.51	66.29	289,400
*Burkholderia cepacia*	BCC0196	Environmental, grass		ERS784922	ERS4267737	GCA_902830615	75 (Illumina)	8.53	66.58	453,915
*Burkholderia ambifaria*	BCC0200	Environmental		ERS785045	ERS4267738	GCA_902829565	76 (Illumina)	7.63	66.55	331,719
*Burkholderia cepacia*	BCC0233	Environmental		ERS785054	ERS4267739	GCA_902830645	66 (Illumina)	8.82	66.50	775,678
*Burkholderia cepacia*	BCC0256	Clinical, sputum	New Zealand	ERS784905	ERS4267740	GCA_902830655	59 (Illumina)	9.19	66.32	987,550
*Burkholderia cepacia*	BCC0367	Clinical		ERS784937	ERS4267741	GCA_902830675	86 (Illumina)	8.68	66.51	796,538
*Burkholderia cepacia*	BCC0970	Environmental		ERS784952	ERS4267742	GCA_902830695	77 (Illumina)	8.37	66.76	428,681
*Burkholderia cepacia*	BCC0974	Environmental		ERS784967	ERS4267743	GCA_902830705	86 (Illumina)	8.59	66.74	450,214
*Burkholderia cepacia*	BCC0977	Environmental		ERS784804	ERS4267744	GCA_902830755	106 (Illumina)	6.93	67.06	573,127
*Burkholderia cepacia*	BCC1058	Environmental		ERS784813	ERS4267745	GCA_902830745	81 (Illumina)	8.37	66.76	505,601
*Burkholderia cepacia*	BCC1273			ERS784829	ERS4267746	GCA_902830735	80 (Illumina)	8.51	66.80	984,555
*Burkholderia* sp.	BCC1640	Environmental	Malaysia	ERS1328852	ERS4267747	GCA_902833125	92 (Illumina)	7.20	66.40	186,730
*Burkholderia* sp.	BCC0044	Clinical, cystic fibrosis patient	UK	ERS785043	ERS4267748	GCA_902832955	70 (Illumina)	8.04	66.82	1,287,212
*Burkholderia* sp.	BCC0322	Clinical, cystic fibrosis patient	UK	ERS784908	ERS4267749	GCA_902833045	71 (Illumina)	7.75	66.27	527,417
*Burkholderia* sp.	BCC0397	Clinical		ERS784940	ERS4267750	GCA_902832865	71 (Illumina)	8.16	66.81	442,581
*Burkholderia* sp.	BCC0398	Environmental		ERS785051	ERS4267751	GCA_902833245	65 (Illumina)	8.01	66.54	387,212
*Burkholderia* sp.	BCC0405	Environmental		ERS784923	ERS4267752	GCA_902833075	71 (Illumina)	7.98	66.08	309,371
*Burkholderia* sp.	BCC0419	Environmental, maize		ERS784958	ERS4267753	GCA_902830285	66 (Illumina)	8.18	66.71	1,058,720
*Burkholderia* sp.	BCC1047	Environmental		ERS784973	ERS4267754	GCA_902833155	83 (Illumina)	7.79	66.79	153,691
*Burkholderia pseudomultivorans*	BCC1191	Clinical		ERS785049	ERS4267755	GCA_902832925	73 (Illumina)	7.55	67.02	243,992
*Burkholderia contaminans*	BCC0123	Clinical, sputum	USA	ERS785009	ERS4267756	GCA_902830725	60 (Illumina)	8.77	66.35	548,891
*Burkholderia contaminans*	BCC0254	Clinical, sputum	Australia	ERS784845	ERS4267757	GCA_902830865	79 (Illumina)	8.35	66.00	564,071
*Burkholderia contaminans*	BCC0339	Clinical		ERS784860	ERS4267758	GCA_902830845	76 (Illumina)	8.29	66.39	1,070,961
*Burkholderia contaminans*	BCC1315	Environmental/industrial		ERS784875	ERS4267759	GCA_902830875	75 (Illumina)	8.02	66.62	652,822
*Burkholderia diffusa*	BCC0106	Clinical, cystic fibrosis patient throat	Canada	ERS785072	ERS4267760	GCA_902830825	72 (Illumina)	7.80	65.92	283,477
*Burkholderia diffusa*	BCC0109	Clinical, respiratory sample	Canada	ERS785028	ERS4267761	GCA_902830855	76 (Illumina)	7.23	66.15	216,537
*Burkholderia diffusa*	LMG 29043	Environmental, soil	USA	ERS4267762	ERS4267762	GCA_902830815	180 (Illumina)	7.15	66.50	236,915
*Burkholderia dolosa*	BCC0306	Clinical	USA	ERS785034	ERS4267763	GCA_902829575	75 (Illumina)	6.50	67.06	519,080
*Burkholderia gladioli*	BCC0238	Clinical, sputum	USA	ERS333128	ERS4267764	GCA_902830885	63 (Illumina)	8.55	68.00	479,899
*Burkholderia gladioli*	BCC0252	Clinical, sputum	Australia	ERS784955	ERS4267765	GCA_902830905	89 (Illumina)	8.22	68.09	289,654
*Burkholderia gladioli*	BCC0507	Clinical, cystic fibrosis patient	Italy	ERS784970	ERS4267766	GCA_902830895	92 (Illumina)	8.36	68.13	335,266
*Burkholderia gladioli*	BCC0771	Environmental, *Gladiolus* sp.	USA	ERS784806	ERS4267767	GCA_902830915	80 (Illumina)	8.80	67.72	967,784
*Burkholderia gladioli*	BCC1317	Environmental/industrial	UK	ERS785017	ERS4267768	GCA_902830925	59 (Illumina)	8.77	67.93	552,562
*Burkholderia gladioli*	BCC1619	Clinical	USA	ERS784817	ERS4267769	GCA_902830945	89 (Illumina)	7.88	68.13	868,935
*Burkholderia gladioli*	BCC1620	Clinical	USA	ERS784832	ERS4267770	GCA_902830985	80 (Illumina)	8.19	68.09	416,357
*Burkholderia gladioli*	BCC1621	Clinical	USA	ERS784849	ERS4267771	GCA_902830995	75 (Illumina)	8.48	67.95	434,143
*Burkholderia gladioli*	BCC1622	Clinical	USA	ERS784864	ERS4267772	GCA_902830955	79 (Illumina)	8.41	68.05	615,150
*Burkholderia gladioli*	BCC1623	Clinical	USA	ERS784879	ERS4267773	GCA_902830935	75 (Illumina)	8.44	67.87	365,793
*Burkholderia gladioli*	BCC1624	Clinical	USA	ERS784895	ERS4267774	GCA_902830975	81 (Illumina)	7.88	68.14	430,243
*Burkholderia gladioli*	BCC1645	Environmental, *Allium cepa* bulb	Australia	ERS784911	ERS4267775	GCA_902830965	73 (Illumina)	8.61	67.87	383,325
*Burkholderia gladioli*	BCC1646	Environmental, *Allium cepa*	India	ERS784928	ERS4267776	GCA_902831005	78 (Illumina)	8.39	67.96	286,552
*Burkholderia gladioli*	BCC1647	Environmental, *Gladiolus* sp.	USA	ERS784943	ERS4267777	GCA_902831075	73 (Illumina)	8.88	67.70	561,180
*Burkholderia gladioli*	BCC1648	Environmental	USA	ERS784957	ERS4267778	GCA_902831035	79 (Illumina)	8.62	67.87	310,631
*Burkholderia gladioli*	BCC1649	Environmental, *Gladiolus* sp.	Zimbabwe	ERS784812	ERS4267779	GCA_902831025	67 (Illumina)	8.25	68.30	554,529
*Burkholderia gladioli*	BCC1650	Environmental, poisoned bongkrek	Indonesia	ERS784828	ERS4267780	GCA_902831015	67 (Illumina)	8.10	68.24	708,245
*Burkholderia gladioli*	BCC1651	Clinical	USA	ERS784844	ERS4267781	GCA_902829385	68 (Illumina)	7.94	68.29	595,470
*Burkholderia gladioli*	BCC1661	Clinical	USA	ERS784861	ERS4267782	GCA_902831055	63 (Illumina)	8.33	68.06	632,785
*Burkholderia gladioli*	BCC1662	Clinical	USA	ERS784878	ERS4267783	GCA_902831065	65 (Illumina)	8.51	68.05	410,006
*Burkholderia gladioli*	BCC1663	Clinical	USA	ERS784894	ERS4267784	GCA_902831085	82 (Illumina)	8.51	68.05	410,015
*Burkholderia gladioli*	BCC1664	Clinical	USA	ERS784910	ERS4267785	GCA_902831045	62 (Illumina)	8.51	68.06	378,407
*Burkholderia gladioli*	BCC1665	Clinical	USA	ERS784925	ERS4267786	GCA_902831105	72 (Illumina)	8.33	68.06	601,921
*Burkholderia gladioli*	BCC1666	Clinical	USA	ERS784942	ERS4267787	GCA_902831115	70 (Illumina)	8.51	68.05	408,038
*Burkholderia gladioli*	BCC1667	Clinical	UK	ERS784960	ERS4267788	GCA_902831095	71 (Illumina)	8.49	67.84	427,447
*Burkholderia gladioli*	BCC1668	Clinical	UK	ERS784974	ERS4267789	GCA_902831165	75 (Illumina)	8.46	67.97	603,154
*Burkholderia gladioli*	BCC1669	Clinical	UK	ERS784982	ERS4267790	GCA_902831175	78 (Illumina)	8.18	68.14	485,051
*Burkholderia gladioli*	BCC1670	Clinical	UK	ERS784814	ERS4267791	GCA_902829325	68 (Illumina)	8.29	68.03	309,426
*Burkholderia gladioli*	BCC1671	Clinical	UK	ERS784830	ERS4267792	GCA_902831225	62 (Illumina)	8.36	68.09	369,886
*Burkholderia gladioli*	BCC1672	Clinical	UK	ERS784846	ERS4267793	GCA_902831265	63 (Illumina)	8.28	68.03	287,015
*Burkholderia gladioli*	BCC1674	Clinical	UK	ERS784994	ERS4267794	GCA_902831195	68 (Illumina)	8.44	67.87	356,821
*Burkholderia gladioli*	BCC1675	Clinical	USA	ERS785062	ERS4267795	GCA_902831215	70 (Illumina)	8.09	68.09	936,495
*Burkholderia gladioli*	BCC1676	Clinical	USA	ERS784863	ERS4267796	GCA_902831315	70 (Illumina)	7.99	68.26	467,101
*Burkholderia gladioli*	BCC1677	Clinical	USA	ERS785071	ERS4267797	GCA_902831155	75 (Illumina)	8.11	68.25	632,433
*Burkholderia gladioli*	BCC1678	Clinical	USA	ERS784880	ERS4267798	GCA_902831285	71 (Illumina)	7.83	68.04	235,084
*Burkholderia gladioli*	BCC1679	Clinical	USA	ERS784896	ERS4267799	GCA_902831135	73 (Illumina)	8.12	68.09	272,665
*Burkholderia gladioli*	BCC1680	Clinical	USA	ERS784912	ERS4267800	GCA_902831205	68 (Illumina)	7.97	68.00	411,881
*Burkholderia gladioli*	BCC1681	Clinical	USA	ERS784927	ERS4267801	GCA_902831305	79 (Illumina)	7.33	68.09	382,283
*Burkholderia gladioli*	BCC1682	Clinical	USA	ERS784944	ERS4267802	GCA_902829635	82 (Illumina)	7.95	68.03	428,648
*Burkholderia gladioli*	BCC1683	Clinical	USA	ERS784962	ERS4267803	GCA_902831235	64 (Illumina)	8.31	68.17	886,986
*Burkholderia gladioli*	BCC1684	Clinical	USA	ERS784975	ERS4267804	GCA_902831185	76 (Illumina)	8.39	67.96	576,064
*Burkholderia gladioli*	BCC1685	Clinical	USA	ERS784983	ERS4267805	GCA_902829495	81 (Illumina)	7.95	68.03	314,381
*Burkholderia gladioli*	BCC1686	Clinical	USA	ERS784816	ERS4267806	GCA_902831255	88 (Illumina)	8.34	68.06	728,975
*Burkholderia gladioli*	BCC1687	Clinical	USA	ERS784833	ERS4267807	GCA_902831145	65 (Illumina)	8.19	68.21	336,464
*Burkholderia gladioli*	BCC1688	Clinical	USA	ERS784848	ERS4267808	GCA_902831125	67 (Illumina)	8.46	68.14	319,266
*Burkholderia gladioli*	BCC1689	Clinical	USA	ERS784865	ERS4267809	GCA_902831275	71 (Illumina)	8.26	67.94	353,209
*Burkholderia gladioli*	BCC1690	Clinical	USA	ERS784881	ERS4267810	GCA_902831295	71 (Illumina)	8.48	67.94	433,041
*Burkholderia gladioli*	BCC1691	Clinical	USA	ERS784898	ERS4267811	GCA_902831245	78 (Illumina)	8.43	68.04	1,964,774
*Burkholderia gladioli*	BCC1692	Clinical	USA	ERS784914	ERS4267812	GCA_902831375	65 (Illumina)	8.39	67.98	810,454
*Burkholderia gladioli*	BCC1693	Clinical	USA	ERS784929	ERS4267813	GCA_902831395	79 (Illumina)	8.22	68.10	668,608
*Burkholderia gladioli*	BCC1694	Clinical	USA	ERS784946	ERS4267814	GCA_902831415	69 (Illumina)	8.48	67.90	688,705
*Burkholderia gladioli*	BCC1695	Clinical	USA	ERS784964	ERS4267815	GCA_902831335	78 (Illumina)	7.91	68.06	538,303
*Burkholderia gladioli*	BCC1696	Clinical	USA	ERS784976	ERS4267816	GCA_902831405	84 (Illumina)	8.28	68.19	429,819
*Burkholderia gladioli*	BCC1697	Clinical	USA	ERS784984	ERS4267817	GCA_902831465	78 (Illumina)	8.09	68.02	652,756
*Burkholderia gladioli*	BCC1698	Clinical	USA	ERS784818	ERS4267818	GCA_902831425	77 (Illumina)	8.50	68.10	434,629
*Burkholderia gladioli*	BCC1699	Clinical	USA	ERS784834	ERS4267819	GCA_902831435	66 (Illumina)	8.35	67.87	354,820
*Burkholderia gladioli*	BCC1700	Clinical	USA	ERS784851	ERS4267820	GCA_902831515	67 (Illumina)	8.49	68.08	1,416,119
*Burkholderia gladioli*	BCC1701	Clinical	USA	ERS784867	ERS4267821	GCA_902829355	67 (Illumina)	8.15	67.98	280,973
*Burkholderia gladioli*	BCC1703	Clinical	USA	ERS785056	ERS4267822	GCA_902831355	64 (Illumina)	8.29	68.22	599,795
*Caballeronia* sp.	BCC1704	Clinical	USA	ERS784900	ERS4267823	GCA_902833455	87 (Illumina)	5.98	63.70	1,316,445
*Burkholderia gladioli*	BCC1705	Clinical	USA	ERS784916	ERS4267824	GCA_902831345	79 (Illumina)	8.15	68.17	551,683
*Caballeronia zhejiangensis*	BCC1706	Clinical	USA	ERS784931	ERS4267825	GCA_902833575	80 (Illumina)	7.02	63.19	368,929
*Burkholderia gladioli*	BCC1707	Clinical	USA	ERS784948	ERS4267826	GCA_902831505	81 (Illumina)	8.01	68.22	534,508
*Burkholderia gladioli*	BCC1708	Clinical	USA	ERS784966	ERS4267827	GCA_902831455	80 (Illumina)	8.34	68.03	945,197
*Burkholderia gladioli*	BCC1709	Clinical	USA	ERS785035	ERS4267828	GCA_902831485	62 (Illumina)	8.47	68.12	414,686
*Burkholderia gladioli*	BCC1710	Clinical	USA	ERS785039	ERS4267829	GCA_902831495	66 (Illumina)	8.38	68.02	615,977
*Burkholderia gladioli*	BCC1711	Clinical	USA	ERS785006	ERS4267830	GCA_902831365	71 (Illumina)	8.35	68.06	335,836
*Burkholderia gladioli*	BCC1712	Clinical	USA	ERS785025	ERS4267831	GCA_902831475	61 (Illumina)	8.21	68.08	388,388
*Burkholderia gladioli*	BCC1713	Clinical	USA	ERS785074	ERS4267832	GCA_902831445	66 (Illumina)	8.47	68.05	147,146
*Burkholderia gladioli*	BCC1714	Clinical	USA	ERS785080	ERS4267833	GCA_902831385	71 (Illumina)	8.50	68.08	361,363
*Burkholderia gladioli*	BCC1715	Clinical	USA	ERS784977	ERS4267834	GCA_902831325	82 (Illumina)	7.97	68.14	954,999
*Burkholderia gladioli*	BCC1716	Clinical	USA	ERS785052	ERS4267835	GCA_902831605	66 (Illumina)	8.50	67.87	385,075
*Burkholderia gladioli*	BCC1717	Clinical	USA	ERS785014	ERS4267836	GCA_902831675	58 (Illumina)	8.43	67.93	430,020
*Burkholderia gladioli*	BCC1719	Clinical	USA	ERS785042	ERS4267837	GCA_902831715	62 (Illumina)	7.99	68.17	429,154
*Burkholderia gladioli*	BCC1720	Clinical	USA	ERS784985	ERS4267838	GCA_902831695	83 (Illumina)	7.91	68.29	770,588
*Burkholderia gladioli*	BCC1721	Clinical	USA	ERS784820	ERS4267839	GCA_902831535	87 (Illumina)	8.22	68.23	1,289,329
*Burkholderia gladioli*	BCC1722	Clinical	USA	ERS784836	ERS4267840	GCA_902831595	71 (Illumina)	8.00	68.18	346,974
*Burkholderia gladioli*	BCC1723	Clinical	USA	ERS784853	ERS4267841	GCA_902831645	69 (Illumina)	8.19	68.25	1,570,053
*Burkholderia gladioli*	BCC1724	Clinical	USA	ERS785060	ERS4267842	GCA_902831545	60 (Illumina)	8.52	68.03	505,438
*Burkholderia gladioli*	BCC1725	Clinical	USA	ERS784869	ERS4267843	GCA_902831555	62 (Illumina)	8.57	68.03	521,790
*Burkholderia gladioli*	BCC1726	Clinical	USA	ERS784885	ERS4267844	GCA_902831525	71 (Illumina)	8.13	68.29	848,284
*Burkholderia gladioli*	BCC1727	Clinical	USA	ERS785068	ERS4267845	GCA_902829345	67 (Illumina)	8.16	68.29	921,754
*Burkholderia gladioli*	BCC1728	Clinical	USA	ERS785003	ERS4267846	GCA_902831685	74 (Illumina)	8.13	68.29	391,569
*Burkholderia gladioli*	BCC1729	Clinical	USA	ERS784902	ERS4267847	GCA_902831705	69 (Illumina)	8.38	67.89	662,719
*Burkholderia gladioli*	BCC1730	Clinical	USA	ERS784917	ERS4267848	GCA_902829545	70 (Illumina)	8.54	68.12	793,173
*Burkholderia gladioli*	BCC1731	Clinical	USA	ERS784997	ERS4267849	GCA_902831635	62 (Illumina)	8.50	67.97	339,104
*Burkholderia gladioli*	BCC1732	Clinical	USA	ERS784933	ERS4267850	GCA_902829585	69 (Illumina)	8.82	67.67	606,420
*Burkholderia gladioli*	BCC1733	Clinical	USA	ERS784951	ERS4267851	GCA_902831575	73 (Illumina)	8.47	68.03	519,208
*Burkholderia gladioli*	BCC1734	Clinical	USA	ERS785079	ERS4267852	GCA_902831585	78 (Illumina)	8.20	68.24	757,647
*Burkholderia gladioli*	BCC1735	Clinical	USA	ERS785008	ERS4267853	GCA_902831665	69 (Illumina)	7.93	67.98	388,060
*Burkholderia gladioli*	BCC1754	Clinical	USA	ERS1371583	ERS4267854	GCA_902829365	64 (Illumina)	8.38	68.20	416,499
*Burkholderia gladioli*	BCC1755	Clinical	USA	ERS1328941	ERS4267855	GCA_902831625	67 (Illumina)	8.27	68.20	324,811
*Burkholderia gladioli*	BCC1756	Clinical	USA	ERS1328753	ERS4267856	GCA_902831655	75 (Illumina)	7.75	68.11	264,066
*Burkholderia gladioli*	BCC1757	Clinical	USA	ERS1371584	ERS4267857	GCA_902831565	70 (Illumina)	8.11	68.25	281,288
*Burkholderia gladioli*	BCC1758	Clinical	USA	ERS1371585	ERS4267858	GCA_902831615	77 (Illumina)	8.42	68.16	347,044
*Burkholderia gladioli*	BCC1759	Clinical	USA	ERS1333276	ERS4267859	GCA_902831785	71 (Illumina)	8.42	67.93	202,663
*Burkholderia gladioli*	BCC1760	Clinical	USA	ERS1371586	ERS4267860	GCA_902831885	74 (Illumina)	8.15	68.21	351,231
*Burkholderia gladioli*	BCC1761	Clinical	USA	ERS1371587	ERS4267861	GCA_902831865	72 (Illumina)	8.11	68.15	227,067
*Burkholderia gladioli*	BCC1762	Clinical	USA	ERS1328756	ERS4267862	GCA_902831745	78 (Illumina)	8.38	67.86	232,045
*Burkholderia gladioli*	BCC1763	Clinical	USA	ERS1328942	ERS4267863	GCA_902831845	70 (Illumina)	8.73	68.08	409,905
*Burkholderia gladioli*	BCC1764	Clinical	USA	ERS1371588	ERS4267864	GCA_902831775	76 (Illumina)	7.95	68.19	273,951
*Burkholderia gladioli*	BCC1765	Clinical	USA	ERS1328883	ERS4267865	GCA_902831855	76 (Illumina)	8.06	68.10	267,531
*Burkholderia gladioli*	BCC1766	Clinical	USA	ERS1371589	ERS4267866	GCA_902831735	77 (Illumina)	8.28	67.89	275,070
*Burkholderia gladioli*	BCC1767	Clinical	USA	ERS1371590	ERS4267867	GCA_902831875	79 (Illumina)	8.15	68.28	365,649
*Burkholderia gladioli*	BCC1768	Clinical	USA	ERS1371591	ERS4267868	GCA_902831905	71 (Illumina)	8.26	68.11	226,248
*Burkholderia gladioli*	BCC1769	Clinical	USA	ERS1328884	ERS4267869	GCA_902831815	67 (Illumina)	8.37	68.02	248,528
*Burkholderia gladioli*	BCC1770	Clinical	USA	ERS1328772	ERS4267870	GCA_902831755	61 (Illumina)	8.42	67.98	261,993
*Burkholderia gladioli*	BCC1771	Clinical	USA	ERS1328943	ERS4267871	GCA_902831805	58 (Illumina)	8.41	67.99	303,762
*Burkholderia gladioli*	BCC1772	Clinical	USA	ERS1328773	ERS4267872	GCA_902831895	58 (Illumina)	8.44	68.03	340,707
*Burkholderia gladioli*	BCC1773	Clinical	USA	ERS1328885	ERS4267873	GCA_902829405	55 (Illumina)	8.08	68.21	264,067
*Burkholderia gladioli*	BCC1774	Clinical	USA	ERS1371592	ERS4267874	GCA_902831835	70 (Illumina)	7.89	68.10	264,045
*Burkholderia gladioli*	BCC1775	Clinical	USA	ERS1333277	ERS4267875	GCA_902831915	65 (Illumina)	8.38	68.13	332,589
*Burkholderia gladioli*	BCC1777	Clinical	USA	ERS1328886	ERS4267877	GCA_902831795	67 (Illumina)	7.93	68.09	275,756
*Burkholderia gladioli*	BCC1778	Clinical	USA	ERS1328776	ERS4267878	GCA_902831825	66 (Illumina)	8.30	68.04	284,651
*Burkholderia gladioli*	BCC1779	Clinical	USA	ERS1328944	ERS4267879	GCA_902831765	65 (Illumina)	8.27	68.02	190,616
*Burkholderia gladioli*	BCC1780	Clinical	USA	ERS1328777	ERS4267880	GCA_902831945	72 (Illumina)	8.45	67.76	220,441
*Burkholderia gladioli*	BCC1781	Clinical	USA	ERS1328887	ERS4267881	GCA_902831975	69 (Illumina)	7.99	68.16	208,905
*Burkholderia gladioli*	BCC1782	Clinical	USA	ERS1328778	ERS4267882	GCA_902829555	62 (Illumina)	8.00	68.24	264,129
*Burkholderia gladioli*	BCC1783	Clinical	USA	ERS1336060	ERS4267883	GCA_902829505	61 (Illumina)	8.39	67.96	224,024
*Burkholderia gladioli*	BCC1784	Clinical	USA	ERS1328888	ERS4267884	GCA_902831925	77 (Illumina)	8.13	68.17	273,802
*Burkholderia gladioli*	BCC1785	Clinical	USA	ERS1328779	ERS4267885	GCA_902831985	75 (Illumina)	7.99	68.26	243,147
*Burkholderia gladioli*	BCC1786	Clinical	USA	ERS1328945	ERS4267886	GCA_902831965	57 (Illumina)	8.33	68.18	347,752
*Burkholderia gladioli*	BCC1787	Clinical	USA	ERS1371593	ERS4267887	GCA_902831955	96 (Illumina)	8.25	68.27	371,296
*Burkholderia gladioli*	BCC1788	Clinical	USA	ERS1371594	ERS4267888	GCA_902831935	68 (Illumina)	8.66	67.84	291,652
*Burkholderia gladioli*	BCC1789	Clinical	USA	ERS1328781	ERS4267889	GCA_902830195	61 (Illumina)	8.33	68.05	274,262
*Burkholderia gladioli*	BCC1790	Clinical	USA	ERS1333278	ERS4267890	GCA_902831995	78 (Illumina)	8.41	67.90	249,452
*Burkholderia gladioli*	BCC1791	Clinical	USA	ERS1328782	ERS4267891	GCA_902832115	68 (Illumina)	8.15	68.14	334,966
*Burkholderia gladioli*	BCC1792	Clinical	USA	ERS1328890	ERS4267892	GCA_902832095	62 (Illumina)	8.13	68.17	276,382
*Burkholderia gladioli*	BCC1793	Clinical	USA	ERS1328783	ERS4267893	GCA_902832155	73 (Illumina)	8.14	68.24	280,591
*Burkholderia gladioli*	BCC1794	Clinical	USA	ERS1328946	ERS4267894	GCA_902832105	70 (Illumina)	8.34	68.23	281,967
*Burkholderia gladioli*	BCC1795	Clinical	USA	ERS1328784	ERS4267895	GCA_902832035	77 (Illumina)	7.82	68.17	275,050
*Burkholderia gladioli*	BCC1796	Clinical	USA	ERS1328891	ERS4267896	GCA_902832055	66 (Illumina)	8.38	68.08	351,151
*Burkholderia gladioli*	BCC1798	Clinical	USA	ERS1371596	ERS4267897	GCA_902832005	75 (Illumina)	8.25	68.07	230,356
*Burkholderia gladioli*	BCC1799	Clinical	USA	ERS1328786	ERS4267898	GCA_902832015	57 (Illumina)	8.11	68.22	343,906
*Burkholderia gladioli*	BCC1800	Clinical	USA	ERS1328892	ERS4267899	GCA_902832165	84 (Illumina)	7.92	68.29	257,500
*Burkholderia gladioli*	BCC1801	Clinical	USA	ERS1328787	ERS4267900	GCA_902832175	75 (Illumina)	7.96	68.24	316,850
*Burkholderia gladioli*	BCC1802	Clinical	USA	ERS1328947	ERS4267901	GCA_902832185	56 (Illumina)	8.45	68.11	270,804
*Burkholderia gladioli*	BCC1803	Clinical	USA	ERS1371597	ERS4267902	GCA_902832025	81 (Illumina)	7.71	68.12	272,468
*Burkholderia gladioli*	BCC1804	Clinical	USA	ERS1371598	ERS4267903	GCA_902832195	71 (Illumina)	8.45	68.00	250,984
*Burkholderia gladioli*	BCC1805	Clinical	USA	ERS1328789	ERS4267904	GCA_902832045	78 (Illumina)	7.86	67.94	270,833
*Burkholderia gladioli*	BCC1806	Clinical	USA	ERS1333279	ERS4267905	GCA_902832075	63 (Illumina)	8.48	68.05	273,269
*Burkholderia gladioli*	BCC1807	Clinical	USA	ERS1371599	ERS4267906	GCA_902832085	69 (Illumina)	8.47	68.00	304,296
*Burkholderia gladioli*	BCC1808	Clinical	USA	ERS1371600	ERS4267907	GCA_902832145	77 (Illumina)	7.97	68.22	293,939
*Burkholderia gladioli*	BCC1809	Clinical	USA	ERS1328791	ERS4267908	GCA_902832125	66 (Illumina)	8.64	67.87	235,923
*Burkholderia gladioli*	BCC1810	Clinical	USA	ERS1328948	ERS4267909	GCA_902830185	64 (Illumina)	8.28	68.22	310,714
*Burkholderia gladioli*	BCC1811	Clinical	USA	ERS1328792	ERS4267910	GCA_902832135	57 (Illumina)	8.61	67.93	305,514
*Burkholderia gladioli*	BCC1812	Clinical	USA	ERS1328895	ERS4267911	GCA_902832065	73 (Illumina)	8.23	67.94	301,688
*Burkholderia gladioli*	BCC1813	Clinical	USA	ERS1328793	ERS4267912	GCA_902832315	65 (Illumina)	8.57	68.01	293,202
*Burkholderia gladioli*	BCC1814	Clinical	USA	ERS1336062	ERS4267913	GCA_902832285	62 (Illumina)	8.21	68.14	206,859
*Burkholderia gladioli*	BCC1815	Clinical	USA	ERS1328794	ERS4267914	GCA_902832295	50 (Illumina)	8.95	67.39	262,346
*Burkholderia gladioli*	BCC1816	Clinical	Canada	ERS1328896	ERS4267915	GCA_902832395	70 (Illumina)	8.18	68.12	326,051
*Burkholderia gladioli*	BCC1817	Clinical	USA	ERS1328795	ERS4267916	GCA_902832305	69 (Illumina)	8.12	68.21	242,936
*Burkholderia gladioli*	BCC1818	Clinical	USA	ERS1333280	ERS4267917	GCA_902832365	56 (Illumina)	8.16	68.23	345,879
*Burkholderia gladioli*	BCC1819	Clinical	USA	ERS1328796	ERS4267918	GCA_902832325	61 (Illumina)	8.42	67.90	331,109
*Burkholderia gladioli*	BCC1820	Clinical	USA	ERS1328897	ERS4267919	GCA_902832245	75 (Illumina)	8.05	68.15	242,112
*Burkholderia gladioli*	BCC1821	Clinical	USA	ERS1328797	ERS4267920	GCA_902829485	76 (Illumina)	8.13	68.08	263,017
*Burkholderia gladioli*	BCC1822	Clinical	USA	ERS1336063	ERS4267921	GCA_902832255	71 (Illumina)	8.08	68.18	402,601
*Burkholderia gladioli*	BCC1823	Clinical	USA	ERS1328798	ERS4267922	GCA_902829525	69 (Illumina)	8.22	68.31	336,871
*Paraburkholderia caribensis*	BCC1824	Clinical	USA	ERS1328898	ERS4267923	GCA_902833585	64 (Illumina)	9.04	62.49	121,444
*Burkholderia gladioli*	BCC1825	Clinical	USA	ERS1328799	ERS4267924	GCA_902832355	70 (Illumina)	8.21	67.96	211,609
*Burkholderia gladioli*	BCC1826	Clinical	USA	ERS1328949	ERS4267925	GCA_902832385	58 (Illumina)	8.38	68.13	338,267
*Burkholderia gladioli*	BCC1827	Clinical	USA	ERS1328800	ERS4267926	GCA_902832345	58 (Illumina)	8.71	67.88	269,894
*Burkholderia gladioli*	BCC1828	Clinical	Canada	ERS1328899	ERS4267927	GCA_902832375	78 (Illumina)	8.24	68.04	268,035
*Burkholderia gladioli*	BCC1829	Clinical	USA	ERS1371601	ERS4267928	GCA_902832235	80 (Illumina)	8.61	67.80	317,992
*Burkholderia gladioli*	BCC1830	Clinical	Canada	ERS1371602	ERS4267929	GCA_902832225	76 (Illumina)	8.40	68.05	419,764
*Burkholderia gladioli*	BCC1831	Clinical	Canada	ERS1371603	ERS4267930	GCA_902832205	74 (Illumina)	8.43	68.00	189,469
*Burkholderia gladioli*	BCC1832	Clinical	USA	ERS1371604	ERS4267931	GCA_902832215	73 (Illumina)	8.16	68.08	208,866
*Burkholderia gladioli*	BCC1833	Clinical	USA	ERS1328803	ERS4267932	GCA_902832275	70 (Illumina)	8.49	68.02	3902,44
*Burkholderia gladioli*	BCC1834	Clinical	USA	ERS1328950	ERS4267933	GCA_902832265	66 (Illumina)	7.90	68.06	286,322
*Burkholderia gladioli*	BCC1835	Clinical	USA	ERS1371605	ERS4267934	GCA_902832335	73 (Illumina)	8.41	67.96	264,845
*Burkholderia gladioli*	BCC1836	Clinical	USA	ERS1328901	ERS4267935	GCA_902832445	63 (Illumina)	8.18	68.30	407,323
*Burkholderia gladioli*	BCC1837	Clinical	USA	ERS1371606	ERS4267936	GCA_902832455	77 (Illumina)	8.14	67.99	206,652
*Burkholderia gladioli*	BCC1838	Clinical	USA	ERS1336064	ERS4267937	GCA_902830215	68 (Illumina)	8.44	67.91	179,624
*Burkholderia gladioli*	BCC1840	Clinical	USA	ERS1328902	ERS4267938	GCA_902832405	71 (Illumina)	8.15	68.14	209,087
*Burkholderia gladioli*	BCC1841	Clinical	USA	ERS1328807	ERS4267939	GCA_902832435	55 (Illumina)	8.72	67.94	274,939
*Burkholderia gladioli*	BCC1842	Clinical	USA	ERS1328951	ERS4267940	GCA_902832415	76 (Illumina)	8.17	68.27	344,808
*Burkholderia gladioli*	BCC1843	Clinical	USA	ERS1328808	ERS4267941	GCA_902832425	67 (Illumina)	8.01	68.10	177,545
*Burkholderia gladioli*	BCC1844	Clinical	USA	ERS1328903	ERS4267942	GCA_902832555	71 (Illumina)	8.20	67.98	219,436
*Burkholderia gladioli*	BCC1845	Clinical	USA	ERS1328809	ERS4267943	GCA_902832535	71 (Illumina)	8.13	68.24	362,515
*Burkholderia gladioli*	BCC1846	Clinical	USA	ERS1371607	ERS4267944	GCA_902832475	68 (Illumina)	8.41	68.00	320,953
*Burkholderia gladioli*	BCC1847	Clinical	USA	ERS1371608	ERS4267945	GCA_902830245	65 (Illumina)	8.41	68.17	287,220
*Burkholderia gladioli*	BCC1848	Clinical	USA	ERS1328904	ERS4267946	GCA_902832525	69 (Illumina)	8.14	68.04	301,164
*Burkholderia gladioli*	BCC1849	Clinical	USA	ERS1371609	ERS4267947	GCA_902832485	94 (Illumina)	8.22	68.29	368,142
*Burkholderia gladioli*	BCC1850	Clinical	USA	ERS1371610	ERS4267948	GCA_902832585	81 (Illumina)	8.53	68.02	275,270
*Burkholderia gladioli*	BCC1851	Clinical	USA	ERS1371611	ERS4267949	GCA_902832495	65 (Illumina)	8.37	68.20	304,291
*Burkholderia gladioli*	BCC1852	Clinical	USA	ERS1371612	ERS4267950	GCA_902832515	72 (Illumina)	8.42	68.05	333,375
*Burkholderia gladioli*	BCC1853	Clinical	USA	ERS1328813	ERS4267951	GCA_902832545	66 (Illumina)	8.22	68.20	326,166
*Burkholderia gladioli*	BCC1854	Clinical	USA	ERS1371613	ERS4267952	GCA_902832465	69 (Illumina)	8.90	67.81	274,690
*Burkholderia gladioli*	BCC1855	Clinical	USA	ERS1371614	ERS4267953	GCA_902832505	64 (Illumina)	8.32	68.01	186,469
*Burkholderia gladioli*	BCC1856	Clinical	USA	ERS1328906	ERS4267954	GCA_902832565	67 (Illumina)	8.05	68.29	286,277
*Burkholderia gladioli*	BCC1857	Clinical	USA	ERS1371615	ERS4267955	GCA_902832595	68 (Illumina)	8.23	68.25	280,379
*Burkholderia gladioli*	BCC1858	Clinical	USA	ERS1371616	ERS4267956	GCA_902832575	54 (Illumina)	8.19	68.22	307,394
*Burkholderia gladioli*	BCC1859	Clinical	USA	ERS1371617	ERS4267957	GCA_902832615	95 (Illumina)	8.26	68.20	334,836
*Burkholderia gladioli*	BCC1860	Clinical	USA	ERS1371618	ERS4267958	GCA_902832655	88 (Illumina)	8.63	68.00	347,760
*Burkholderia gladioli*	BCC1861	Clinical	USA	ERS1371619	ERS4267959	GCA_902829615	79 (Illumina)	8.63	67.75	216,674
*Burkholderia gladioli*	BCC1862	Clinical	USA	ERS1371620	ERS4267960	GCA_902832685	89 (Illumina)	8.38	68.14	294,407
*Burkholderia gladioli*	BCC1863	Clinical	USA	ERS1371621	ERS4267961	GCA_902832605	74 (Illumina)	8.27	68.23	294,023
*Burkholderia gladioli*	BCC1864	Clinical	USA	ERS1328908	ERS4267962	GCA_902830235	53 (Illumina)	8.50	67.90	212,901
*Burkholderia gladioli*	BCC1865	Clinical	USA	ERS1328819	ERS4267963	GCA_902832725	72 (Illumina)	8.23	68.15	233,312
*Burkholderia gladioli*	BCC1866	Clinical	USA	ERS1371622	ERS4267964	GCA_902832695	71 (Illumina)	8.46	67.99	287,257
*Burkholderia gladioli*	BCC1867	Clinical	USA	ERS1371623	ERS4267965	GCA_902832675	72 (Illumina)	8.40	67.96	178,703
*Burkholderia gladioli*	BCC1868	Clinical	USA	ERS1371624	ERS4267966	GCA_902832715	62 (Illumina)	7.93	68.19	393,583
*Burkholderia gladioli*	BCC1869	Clinical	USA	ERS1328821	ERS4267967	GCA_902832665	64 (Illumina)	8.60	67.92	311,968
*Burkholderia gladioli*	BCC1870	Clinical	USA	ERS1371625	ERS4267968	GCA_902832645	86 (Illumina)	8.35	67.95	186,380
*Burkholderia gladioli*	BCC1871	Clinical	USA	ERS1371626	ERS4267969	GCA_902832705	95 (Illumina)	8.06	68.11	284,538
*Burkholderia gladioli*	BCC1872	Clinical	USA	ERS1371627	ERS4267970	GCA_902832745	74 (Illumina)	8.43	68.15	286,407
*Burkholderia gladioli*	BCC1880			ERS1371628	ERS4267971	GCA_902832635	73 (Illumina)	8.23	68.04	621,809
*Burkholderia gladioli*	BCC1881			ERS1371629	ERS4267972	GCA_902832735	71 (Illumina)	8.39	68.20	350,910
*Burkholderia gladioli*	BCC1882			ERS1371630	ERS4267973	GCA_902832625	58 (Illumina)	8.39	68.20	350,910
*Burkholderia gladioli*	BCC1883			ERS1371631	ERS4267974	GCA_902832785	71 (Illumina)	8.13	68.31	603,194
*Burkholderia glumae*	LMG 2196	Environmental, *Oryza sativa*	Japan	ERS784969	ERS4267975	GCA_902832765	92 (Illumina)	6.66	68.35	162,494
*Burkholderia lata*	BCC0174	Environmental, soil	Trinidad and Tobago	ERS785040	ERS4267976	GCA_902832775	63 (Illumina)	8.48	66.57	1,023,694
*Burkholderia* sp.	BCC0217	Clinical, cystic fibrosis patient	USA	ERS784822	ERS4267977	GCA_902833005	74 (Illumina)	9.44	65.63	453,341
*Burkholderia latens*	BCC1625	Clinical, cystic fibrosis patient	Italy	ERS784839	ERS4267978	GCA_902832795	80 (Illumina)	7.08	66.83	469,972
*Burkholderia latens*	BCC1626	Clinical, cystic fibrosis patient	UK	ERS784855	ERS4267979	GCA_902832755	78 (Illumina)	7.25	66.69	638,063
*Burkholderia metallica*	BCC0095	Clinical, sputum	Canada	ERS785019	ERS4267980	GCA_902832815	67 (Illumina)	7.75	66.76	474,034
*Burkholderia metallica*	BCC0483	Clinical		ERS784872	ERS4267981	GCA_902832805	75 (Illumina)	7.55	67.06	469,713
*Burkholderia metallica*	LMG 24068	Clinical, cystic fibrosis patient	USA	ERS784887	ERS4267982	GCA_902832845	75 (Illumina)	7.55	67.06	476,026
*Burkholderia multivorans*	BCC0005	Clinical, sputum	Canada	ERS785011	ERS4267983	GCA_902832835	87 (Illumina)	6.39	67.37	1,402,971
*Burkholderia multivorans*	BCC0008	Clinical, sputum	UK	ERS784904	ERS4267984	GCA_902832825	88 (Illumina)	6.63	67.18	793,813
*Burkholderia multivorans*	BCC0010	Clinical, non-cystic fibrosis patient brain abscess	UK	ERS784919	ERS4267985	GCA_902833875	92 (Illumina)	6.27	67.24	486,046
*Burkholderia multivorans*	BCC0031	Clinical, sputum	Canada	ERS785002	ERS4267986	GCA_902833805	89 (Illumina)	6.53	67.07	561,216
*Burkholderia multivorans*	BCC0037	Clinical, chronic granulomatous disease, lung	USA	ERS785053	ERS4267987	GCA_902833915	93 (Illumina)	6.56	67.16	303,874
*Burkholderia multivorans*	BCC0050	Environmental, soil	UK	ERS785036	ERS4267988	GCA_902833885	78 (Illumina)	6.97	66.86	531,523
*Burkholderia multivorans*	BCC0059	Clinical, sputum	Canada	ERS785022	ERS4267989	GCA_902833835	74 (Illumina)	6.76	67.09	384,200
*Burkholderia multivorans*	BCC0067	Clinical, sputum	Canada	ERS784935	ERS4267990	GCA_902833855	96 (Illumina)	6.12	67.36	349,267
*Burkholderia multivorans*	BCC0089	Clinical, sputum	Canada	ERS785033	ERS4267991	GCA_902833895	82 (Illumina)	6.39	67.21	595,797
*Burkholderia multivorans*	BCC0093	Clinical, respiratory sample	Canada	ERS785061	ERS4267992	GCA_902832975	96 (Illumina)	6.30	67.22	342,629
*Burkholderia multivorans*	BCC0099	Clinical, respiratory sample	Canada	ERS785069	ERS4267993	GCA_902832855	94 (Illumina)	6.93	66.81	276,171
*Burkholderia multivorans*	BCC0102	Clinical, respiratory sample	Canada	ERS784954	ERS4267994	GCA_902833015	80 (Illumina)	6.66	67.18	459,105
*Burkholderia multivorans*	BCC0115	Clinical, sputum	USA	ERS784971	ERS4267995	GCA_902830305	102 (Illumina)	6.22	67.36	413,909
*Burkholderia multivorans*	BCC0149	Clinical, cystic fibrosis patient	USA	ERS785050	ERS4267996	GCA_902832965	86 (Illumina)	6.35	67.17	436,404
*Burkholderia multivorans*	BCC0175	Clinical, chronic granulomatous disease, blood	USA	ERS785024	ERS4267997	GCA_902832875	77 (Illumina)	6.71	66.72	328,297
*Burkholderia multivorans*	BCC0244	Clinical, sputum	Canada	ERS785004	ERS4267998	GCA_902832915	93 (Illumina)	6.57	67.18	435,992
*Burkholderia oklahomensis*	LMG 23618	Environmental		ERS784979	ERS4267999	GCA_902829515	88 (Illumina)	7.09	67.11	364,599
*Burkholderia plantarii*	LMG 9035	Environmental, *Oryza sativa* cv. Koshihikari, seedling with blight and chlorosis	Japan	ERS784824	ERS4268000	GCA_902832905	79 (Illumina)	8.04	68.61	305,422
*Burkholderia pyrrocinia*	BCC0171	Environmental, cornfield soil	USA	ERS785059	ERS4268001	GCA_902832995	66 (Illumina)	8.16	66.18	325,972
*Burkholderia pyrrocinia*	LMG 14191	Environmental, soil		ERS785083	ERS4268002	GCA_902832895	75 (Illumina)	7.86	66.51	520,567
*Burkholderia pyrrocinia*	BCC0986	Environmental		ERS784857	ERS4268003	GCA_902829605	74 (Illumina)	7.60	66.70	556,419
*Burkholderia pyrrocinia*	BCC1346	Environmental		ERS784841	ERS4268004	GCA_902832935	70 (Illumina)	8.34	65.94	282,972
*Burkholderia pyrrocinia*	BCC1364	Clinical		ERS784874	ERS4268005	GCA_902832945	75 (Illumina)	7.62	66.57	587,939
*Burkholderia seminalis*	LMG 24067	Clinical, cystic fibrosis patient	USA	ERS784889	ERS4268006	GCA_902832885	70 (Illumina)	7.97	67.09	523,234
*Burkholderia seminalis*	BCC1628	Environmental, *Oryza sativa* seed	Philippines	ERS784906	ERS4268007	GCA_902833035	60 (Illumina)	8.24	66.71	331,981
*Burkholderia* sp.	BCC1642	Environmental		ERS1328853	ERS4268008	GCA_902829715	78 (Illumina)	9.22	62.08	159,099
*Burkholderia stabilis*	BCC0186	Clinical, cystic fibrosis patient	Belgium	ERS785081	ERS4268009	GCA_902833135	92 (Illumina)	7.15	66.55	267,680
*Burkholderia stabilis*	BCC0237	Environmental, albuterol inhaler	USA	ERS785013	ERS4268010	GCA_902833115	58 (Illumina)	8.52	66.46	323,418
*Burkholderia stabilis*	BCC0285	Clinical, infected tissue of tibial fracture		ERS784949	ERS4268011	GCA_902833105	80 (Illumina)	8.75	66.23	274,531
*Burkholderia stabilis*	BCC1308	Environmental/industrial		ERS784921	ERS4268012	GCA_902833185	61 (Illumina)	8.25	66.51	685,734
*Burkholderia stagnalis*	LMG 28156	Environmental, soil	Australia	ERS1371647	ERS4268013	GCA_902830275	66 (Illumina)	8.03	67.24	262,379
*Burkholderia territorii*	LMG 28158	Environmental, water	Australia	ERS1328858	ERS4268014	GCA_902833055	92 (Illumina)	6.98	66.66	277,911
*Burkholderia ubonensis*	LMG 20358	Environmental, surface soil	Thailand	ERS784956	ERS4268015	GCA_902833085	66 (Illumina)	7.77	67.30	623,290
*Burkholderia vietnamiensis*	BCC0027	Clinical, cystic fibrosis patient	USA	ERS785078	ERS4268016	GCA_902833235	85 (Illumina)	6.87	66.94	283,796
*Burkholderia vietnamiensis*	BCC0028	Clinical, cystic fibrosis patient	Sweden	ERS785077	ERS4268017	GCA_902833215	94 (Illumina)	6.67	67.25	195,580
*Burkholderia vietnamiensis*	BCC0029	Clinical, chronic granulomatous disease, lung abscess	Canada	ERS784991	ERS4268018	GCA_902829455	95 (Illumina)	6.98	66.73	204,529
*Burkholderia vietnamiensis*	LMG 10929	Environmental, *Oryza sativa* rhizosphere soil	Vietnam	ERS785016	ERS4268019	GCA_902830295	75 (Illumina)	6.88	66.90	308,470
*Burkholderia vietnamiensis*	BCC0040	Clinical, non-cystic fibrosis patient		ERS784999	ERS4268020	GCA_902833385	89 (Illumina)	6.26	66.68	397,866
*Burkholderia vietnamiensis*	BCC0042	Environmental, hospital		ERS784972	ERS4268021	GCA_902833285	105 (Illumina)	6.29	66.65	667,227
*Burkholderia vietnamiensis*	BCC0046	Clinical, non-cystic fibrosis patient		ERS785044	ERS4268022	GCA_902833275	81 (Illumina)	6.77	66.97	511,799
*Burkholderia vietnamiensis*	BCC0104	Clinical, lymph node	USA	ERS785007	ERS4268023	GCA_902833335	74 (Illumina)	7.06	66.85	228,863
*Burkholderia vietnamiensis*	BCC0124	Clinical, sputum	USA	ERS785030	ERS4268024	GCA_902833355	73 (Illumina)	7.11	66.93	309,681
*Burkholderia vietnamiensis*	BCC0126	Clinical, sputum	USA	ERS1328823	ERS4268025	GCA_902833375	72 (Illumina)	6.81	67.10	186,133
*Burkholderia vietnamiensis*	BCC0128	Clinical, sputum	Canada	ERS784980	ERS4268026	GCA_902833415	96 (Illumina)	6.63	67.17	400,883
*Burkholderia vietnamiensis*	BCC0129	Clinical	USA	ERS1328846	ERS4268027	GCA_902833445	79 (Illumina)	6.98	66.46	186,188
*Burkholderia vietnamiensis*	BCC0136	Clinical, throat	Canada	ERS785029	ERS4268028	GCA_902833435	69 (Illumina)	7.29	66.68	225,055
*Burkholderia vietnamiensis*	BCC0142	Clinical	USA	ERS784988	ERS4268029	GCA_902833265	86 (Illumina)	7.21	66.73	246,894
*Burkholderia vietnamiensis*	BCC0151	Clinical	USA	ERS784826	ERS4268030	GCA_902833395	95 (Illumina)	6.08	67.33	168,450
*Burkholderia vietnamiensis*	BCC0157	Clinical, sputum	USA	ERS1371645	ERS4268031	GCA_902833295	102 (Illumina)	6.35	67.18	137,809
*Burkholderia vietnamiensis*	BCC0185	Clinical, neck abscess	Chile	ERS1336070	ERS4268032	GCA_902833315	77 (Illumina)	7.12	66.80	202,960
*Burkholderia vietnamiensis*	BCC0190	Clinical	USA	ERS785015	ERS4268033	GCA_902830265	84 (Illumina)	6.16	66.76	523,281
*Burkholderia vietnamiensis*	BCC0194	Environmental, soil		ERS785075	ERS4268034	GCA_902833365	75 (Illumina)	7.06	66.93	158,113
*Burkholderia vietnamiensis*	BCC0324	Clinical, non-cystic fibrosis patient		ERS784843	ERS4268035	GCA_902833345	92 (Illumina)	6.21	66.62	311,192
*Burkholderia vietnamiensis*	BCC0587	Environmental, soil		ERS1328927	ERS4268037	GCA_902833305	71 (Illumina)	7.39	66.79	187,227
*Burkholderia vietnamiensis*	BCC1161	Clinical		ERS785038	ERS4268038	GCA_902833255	78 (Illumina)	6.62	67.33	306,697
*Burkholderia vietnamiensis*	BCC1162	Clinical		ERS784859	ERS4268039	GCA_902833325	85 (Illumina)	6.62	67.33	308,591
*Burkholderia vietnamiensis*	BCC1163	Clinical		ERS784876	ERS4268040	GCA_902833545	81 (Illumina)	6.62	67.33	314,533
*Burkholderia vietnamiensis*	BCC1170	Clinical		ERS785023	ERS4268041	GCA_902833495	79 (Illumina)	6.91	66.39	157,040
*Burkholderia vietnamiensis*	BCC1172	Environmental		ERS785031	ERS4268042	GCA_902833625	71 (Illumina)	7.39	66.30	161,339
*Burkholderia vietnamiensis*	BCC1176	Clinical		ERS785046	ERS4268043	GCA_902833605	76 (Illumina)	7.09	66.83	457,880
*Burkholderia vietnamiensis*	BCC1186	Clinical		ERS784891	ERS4268044	GCA_902833535	72 (Illumina)	7.08	66.45	138,269
*Burkholderia vietnamiensis*	BCC1193	Clinical		ERS785058	ERS4268045	GCA_902833555	72 (Illumina)	7.25	66.29	160,650
*Burkholderia vietnamiensis*	BCC1301	Environmental/industrial		ERS785065	ERS4268046	GCA_902833615	80 (Illumina)	7.05	66.78	246,898
*Burkholderia vietnamiensis*	BCC1304	Environmental/industrial		ERS784990	ERS4268047	GCA_902833475	71 (Illumina)	8.09	66.11	246,358
*Burkholderia vietnamiensis*	BCC1309	Environmental/industrial		ERS785063	ERS4268048	GCA_902833565	82 (Illumina)	7.08	66.91	276,656
*Burkholderia vietnamiensis*	BCC1409	Environmental/industrial		ERS785012	ERS4268049	GCA_902833595	72 (Illumina)	7.22	66.67	308,880
*Burkholderia vietnamiensis*	BCC1412	Environmental/industrial		ERS785020	ERS4268050	GCA_902833465	71 (Illumina)	7.34	66.61	178,163
*Burkholderia cepacia*	BCC1633	Environmental	Malaysia	ERS1328923	ERS4268051	GCA_902830795	81 (Illumina)	8.84	66.29	275,215
*Caballeronia glathe*i	BCC0772	Environmental, lateritic soil	Germany	ERS784993	ERS4268052	GCA_902833485	75 (Illumina)	8.74	64.38	262,729
*Paraburkholderia bryophila*	BCC1875	Environmental, isolated from *Sphagnum rubellum* moss	Germany	ERS1328963	ERS4268053	GCA_902833525	71 (Illumina)	8.09	62.91	200,899
*Paraburkholderia* sp.	BCC1876	Environmental, isolated from *Aulacomnium palustre* moss	Germany	ERS1328856	ERS4268054	GCA_902833695	68 (Illumina)	8.50	62.75	785,957
*Paraburkholderia caledonica*	LMG 19076	Environmental, soil rhizosphere	UK	ERS784802	ERS4268055	GCA_902833635	99 (Illumina)	7.31	61.96	449,131
*Paraburkholderia caribensis*	LMG 18531	Environmental, soil, vertisol		ERS784810	ERS4268056	GCA_902833515	66 (Illumina)	8.97	62.60	301,636
*Paraburkholderia fungorum*	LMG 16225	Environmental, *Phanerochaete chrysosporium* fungus	France	ERS784892	ERS4268057	GCA_902833645	67 (Illumina)	8.93	61.85	451,759
*Paraburkholderia hospit*a	LMG 20598	Environmental, agricultural soil	Belgium	ERS784978	ERS4268058	GCA_902833685	56 (Illumina)	11.25	61.88	202,916
*Paraburkholderia kururiensis*	LMG 19447	Environmental, aquifer	Japan	ERS784986	ERS4268059	GCA_902833705	84 (Illumina)	7.54	64.83	257,717
*Burkholderia vietnamiensis*	BCC1878	Environmental, isolated from *Aulacomnium palustre* moss	Germany	ERS1328857	ERS4268060	GCA_902829795	91 (Illumina)	7.03	66.73	129,098
*Paraburkholderia mimosarum*	LMG 23256	Environmental, *Mimosa pigra* root nodule	China	ERS785026	ERS4268061	GCA_902833725	60 (Illumina)	8.60	63.84	198,630
*Paraburkholderia phenazinium*	BCC1873	Environmental		ERS1328926	ERS4268062	GCA_902829745	70 (Illumina)	8.96	62.26	287,222
*Paraburkholderia phenazinium*	BCC1874			ERS1328855	ERS4268063	GCA_902833675	71 (Illumina)	9.00	62.28	309,756
*Paraburkholderia phenoliruptrix*	LMG 22037	Environmental, chemostat		ERS1371595	ERS4268064	GCA_902833655	82 (Illumina)	7.36	63.48	174,050
*Paraburkholderia phymatum*	LMG 21445	Environmental, *Machaerium lunatum* root nodule	French Guiana	ERS784987	ERS4268065	GCA_902833665	72 (Illumina)	8.60	62.32	334,756
*Paraburkholderia sacchari*	LMG 19450	Environmental, sugarcane plantation soil	Brazil	ERS785005	ERS4268066	GCA_902833715	81 (Illumina)	7.27	64.04	428,857
*Paraburkholderia terricola*	LMG 20594	Environmental, agricultural soil	Belgium	ERS785021	ERS4268067	GCA_902833815	67 (Illumina)	7.34	63.65	179,029
*Paraburkholderia tropica*	LMG 22274	Environmental, sugarcane roots	Brazil	ERS784938	ERS4268068	GCA_902833865	60 (Illumina)	8.60	64.77	492,153
*Paraburkholderia tropica*	BCC1637	Environmental	Malaysia	ERS1328924	ERS4268069	GCA_902833825	80 (Illumina)	8.35	64.80	300,108
*Paraburkholderia tuberum*	LMG 21444	Environmental, *Aspalathus carnosa* root nodule	South Africa	ERS785070	ERS4268070	GCA_902833905	64 (Illumina)	8.79	63.06	137,501
*Robbsia andropogonis*	LMG 2129	Environmental, *Sorghum bicolor*	USA	ERS784901	ERS4268071	GCA_902833845	84 (Illumina)	6.33	58.87	340,675
*Trinickia caryophylli*	LMG 2155	Environmental, *Dianthus caryophyllus*	USA	ERS784825	ERS4268072	GCA_902833925	104 (Illumina)	6.56	64.73	645,930
*Burkholderia stagnalis*	BCC1350	Environmental			ERS4268073	GCA_902833205	150 (PacBio)	8.45	67.06	3,279,208
*Burkholderia gladioli*	BCC0238	Clinical, sputum	USA		ERS4268074	GCA_902829535	150 (PacBio)	8.64	67.94	4,046,521
*Burkholderia vietnamiensis*	BCC0268	Environmental, soil	New Zealand		ERS4268075	GCA_902833425	150 (PacBio)	6.81	67.10	2,221,444
*Burkholderia vietnamiensis*	BCC1408	Environmental/industrial, diesel contaminant	UK		ERS4268076	GCA_902833505	150 (PacBio)	7.89	66.39	1,872,953

The genome collection contains 24 named *Burkholderia* species and 17 genomes that possessed an ANI value of <95% compared to sequenced type strains. Within the Burkholderia cepacia complex, the first representative genome of Burkholderia arboris has been deposited, as well as genomes for type strains of Burkholderia anthina, Burkholderia metallica, Burkholderia seminalis, Burkholderia territorii, and Burkholderia ubonensis. The collection considerably increases the number of curated *Burkholderia* genomes, specifically for Burkholderia gladioli (207 genomes), Burkholderia ambifaria (60 genomes), and Burkholderia vietnamiensis (35 genomes).

The *Paraburkholderia* genomes cover 14 named species and 1 unnamed species based on ANI analysis. The type strains of Paraburkholderia caledonica and Paraburkholderia tuberum were sequenced, and the type strains of 11 species were resequenced. Additional genome assemblies were deposited for Paraburkholderia megapolitana and Paraburkholderia phenazinium. Finally, three *Caballeronia* genomes were identified in the collection, i.e., Caballeronia glathei, Caballeronia zhejiangensis, and an uncharacterized *Caballeronia* species. The type strains of Robbsia andropogonis and Trinickia caryophylli were also resequenced.

Multiple novel metabolites have been identified, and their biosynthetic pathways have been elucidated, including the antimycobacterial compound gladiolin (9) and the lipopeptidiolide antibiotic icosalides (15) in B. gladioli. The discovery and biosynthesis of enacyloxin lla, an antibiotic with potent activity against Acinetobacter baumannii (16–18), and the antioomycete metabolite cepacin (19) have been studied in B. ambifaria. Further analysis of this *Burkholderiaceae* genome resource is under way to identify additional biologically active specialized metabolites and their potential applications.

### Data availability.

The genome sequences in this announcement have been deposited in the European Nucleotide Archive (ENA) under the project accession number PRJEB35318. The BioSample accession numbers for the 454 genome assemblies are ERS4267623 to ERS4268076. The Illumina paired-end read data associated with these genome assemblies have been deposited under the project accession number PRJEB9765, with the exception of Burkholderia diffusa LMG 29043, which is available under project accession number PRJEB35318.
